# Feel-Own-Move: a psychomotor therapy program for victims of intimate partner violence living in shelter homes. Feasibility and effects on mental health, bodily dissociation, and quality of life

**DOI:** 10.3389/fpsyg.2023.1154385

**Published:** 2023-07-06

**Authors:** Joana Machorrinho, José Marmeleira, Guida Veiga, Graça Duarte Santos

**Affiliations:** ^1^Comprehensive Health Research Center, Universidade de Évora, Évora, Portugal; ^2^Departamento de Desporto e Saúde, Escola de Saúde e Desenvolvimento Humano, Universidade de Évora, Évora, Portugal

**Keywords:** psychomotor therapy, intimate partner violence, women, health, quality of life, bodily dissociation, intervention

## Abstract

**Introduction:**

Intimate partner violence (IPV) is a worldwide concern, impacting victims’ mental health, physical health, and quality of life. High rates of posttraumatic stress disorder (PTSD), depression, anxiety, bodily dissociation, and somatic symptoms have been found in victims of IPV, with an important impact on the chronicity of impairments and on the outcomes of psychological interventions. Therapeutic interventions available in shelter homes for victims are scarce in addressing their body–mind needs therefore asking for better empirical research. Thus, the aim of this study was to evaluate the feasibility and effects of Feel-Own-Move (FOM), an 8-week psychomotor therapy program for victims of IPV, on their mental health, levels of bodily dissociation, and general quality of life.

**Methods:**

A within-subject repeated measures design was used to evaluate the intervention effects, and feasibility results were analyzed.

**Results:**

Seventeen women completed the program (mean age 42.8 years, range 21–64). Results showed a significant decrease in levels of bodily dissociation, with FOM having a large effect size. The intervention also had a large effect size at increasing the environment domain of quality of life, although no statistically significant differences were found. FOM ended with excellent rates of reach, adherence, acceptability, and satisfaction. A positive retention rate was also found.

**Discussion:**

In conclusion, FOM seems to be a feasible psychomotor therapy intervention for female victims of IPV living in shelters. Importantly, this program showed to be effective in reducing bodily dissociation among participants, which is suggested to prospectively contribute to their mental health and quality of life.

## Introduction

Intimate partner violence (IPV) refers to any physical, psychological, sexual or economic act of violence perpetrated to a victim in the context of an intimate relationship (World Health Organization, 2021). IPV is a widespread social concern that affects about one-third of women worldwide (World Health Organization, 2021). Victims extensively report symptoms of anxiety, depression, posttraumatic stress (PTSD), and altered patterns of sleep and eating (Lagdon et al., 2014). Research has also found a high prevalence of reported pain, neuromuscular, and gastrointestinal symptoms, which were associated with the severity of symptoms of PTSD and depression, and with health-related quality of life (Kelly, 2010).

Mental health, physical health, and behavior are intertwined. Some of the internal processes necessary for self-regulation, knowledge, and self-growth, rely on information arouse from the psychophysical awareness, namely the awareness of bodily sensations and the connection to the body (Price, 2007). Aligned with this, an alarming prevalence of self-injury and suicidal behaviors, along with symptoms of body disownership [the sense of not owning one’s body (Ataria, 2018)] and bodily dissociation [the feeling of being separate from one’s body and emotions; an “avoidance of internal experience” (Price, 2007)] have been found among victims of IPV (Machorrinho et al., 2021a,b). Importantly, body disownership and bodily dissociation have been pointed out as responsible for the development of PTSD symptoms, the restriction of treatment outcomes, and the prognoses of IPV victims (Ataria, 2018; Tschoeke et al., 2019). Bodily dissociation can include difficulty with the identification and expression of emotions, and also represents a risk factor for the development of anxiety and depression symptoms in female victims of IPV (Machorrinho et al., 2021b). Dissociation often emerges as a defense psychological mechanism, to protect the bodily self and to cope with pain and trauma (Price, 2007). It has a particularly higher prevalence among victims of physical and sexual IPV, for whom it is negatively associated with health-related quality of life (Costa et al., 2015).

Support centers, shelter homes and primary social and health care services strive to increase the reach and effectiveness of programs to prevent IPV and reduce its consequences (Arroyo et al., 2017). Shelter homes (also called transitional supportive housing, TSH) represents a tertiary prevention strategy that aims to reduce mortality or disability (Coker, 2004). In this regard, shelters commonly deliver advocacy support, Cognitive Behavioral Therapy (CBT) and social assistance while providing a safe home, food, education, and employment opportunities for a limited period (6–12 months; Klein et al., 2021). By being physically secure and distant from the violent environment, shelter homes might represent a valuable place for therapeutic interventions targeted at victims’ recovery of health and reconstruction of life and identity (Arroyo et al., 2017). Although some advocacy and psychoeducation interventions have shown positive effects, mostly on healthcare use and mental health symptoms, the broad and complex consequences of IPV on women’s health and identity require extensive research and consideration of body–mind interventions (Eckhardt et al., 2013; Arroyo et al., 2017; Ogbe et al., 2020).

The lasting effects of trauma on the body and on the body–mind relationship, have brought growing interest to the development of effective therapeutic interventions (Classen et al., 2021). Traumatic experiences dysregulate neurochemical and psychophysiological usual responses to stress (Shepherd and Wild, 2014; Payne et al., 2015; Van der Kolk, 2015). In victims of recurrent violence and stress, among the major consequences of this chronic dysregulation, desensitization to stress triggers and numbing, or hyper-arousal chronic states have been reported as two possible extreme responses (Van der Kolk, 2015; Van de Kamp and Hoven, 2019). Hence, increasing research recommends a bottom-up approach as a starting point in the therapeutic work with trauma victims to facilitate arousal and affect regulation (Ogden et al., 2006; Van der Kolk, 2015; Van de Kamp and Hoven, 2019). Diverse body–mind-oriented interventions have shown moderate to large effects on decreasing PTSD symptomatology in adults with diverse trauma origins (Van de Kamp et al., 2019). Examples can be Yoga (Van der Kolk, 2015), Sensorimotor psychotherapy (Classen et al., 2021), Somatic Experiencing (Payne et al., 2015; Andersen et al., 2017), Dance/movement therapy (Dieterich-Hartwell, 2017) and Psychomotor therapy (PmT; Bieleveldt, 2019; Rekkers et al., 2021). These approaches reclaim bottom-up sensations and regulation processes to address traumatic imprints and regulate arousal (Van de Kamp et al., 2019).

PmT is a movement and body-oriented therapy that provides that bottom-up approach and explores embodied emotional, cognitive and relational identity processes (European Forum of Psychomotricity, 2012). PmT acts upon complex bodily dimensions, namely the real body, the imaginary body, the functional body, the body schema and the body image (Potel, 2019; Fernandes et al., 2022). The awareness and processing of those bodily dimensions are promoted through movement, breathing, and self-expression, as a vehicle to enhance the adaptive functioning of the individual (Llaurado, 2008; Lebre et al., 2020). Distinctively, solving problematic behaviors (namely disruptive behaviors) is not the primary goal for a PmT therapist. Instead, he/she works at the expression of anguishes embedded in the body, allowing for new representations of the Self, the others, and the world, with an indirect impact on behavior (Emck and Scheffers, 2019; Lebre et al., 2020; Marmeleira et al., 2023).

The psychomotor therapist has a complex graduated training (as part of the bachelor’s degree in psychomotricity) that allows him/her an in-depth experience in the holistic understanding and facilitation of bodily expression and movement, as well as promoting body awareness and regulating arousal. His/her therapeutic accompaniment is characterized by a consistent, comprehensive, responsive, and encompassing attitude (Llaurado, 2008; Potel, 2019), also revealed at the level of resonance and kinesthetic empathy. These principles and attitudes are the basis of an approach that is proposed to be consistent in the work on revalidating sensations and supporting the participants’ verbal and non-verbal expression (Llaurado, 2008).

Feel-Own-Move (FOM) is a PmT program specifically designed to be implemented in shelter homes, and its therapeutic mechanisms have been recently described (Marmeleira et al., 2023). Upon a safe, empathic and cohesive investment of the therapeutic space and of the therapeutic relationship, participants are invited to (i) experience interoceptive and proprioceptive sensations, (ii) become aware of bodily internal sensations and representations with a non-judgmental approach, (iii) experience and learn relaxation techniques, and (iv) express, through movement, writing, drawing and verbal and non-verbal communication, their body knowledge, insecurity, fears and desires (Caldwell and Victoria, 2011; Payne et al., 2015; Van de Kamp and Hoven, 2019; Björkman and Ekblom, 2022; Machorrinho et al., 2022; Marmeleira et al., 2023). FOM thus allows victims of IPV the safe embodiment of new internal and external representations.

FOM is an 8-week PmT program that integrates the benefits of group sessions and individual sessions, apart from being implemented in a short period of time. It considers the minimum time needed for a therapeutic process to occur, and the short periods of time that most victims actually stay in the shelter home (Arroyo et al., 2017). However, the effectiveness and feasibility of FOM was not empirically examined yet. In this regard, the aim of the present study was twofold. First, the feasibility (reach, adherence, retention and acceptability) of FOM was assessed in a sample of female victims of IPV living in three different shelter homes. Second, the effects of FOM on quality of life and mental health indicators, such as bodily dissociation, anxiety, depression and PTSD of women victims of IPV living in shelters was examined.

## Methods

### Study design

**Figure 1:**
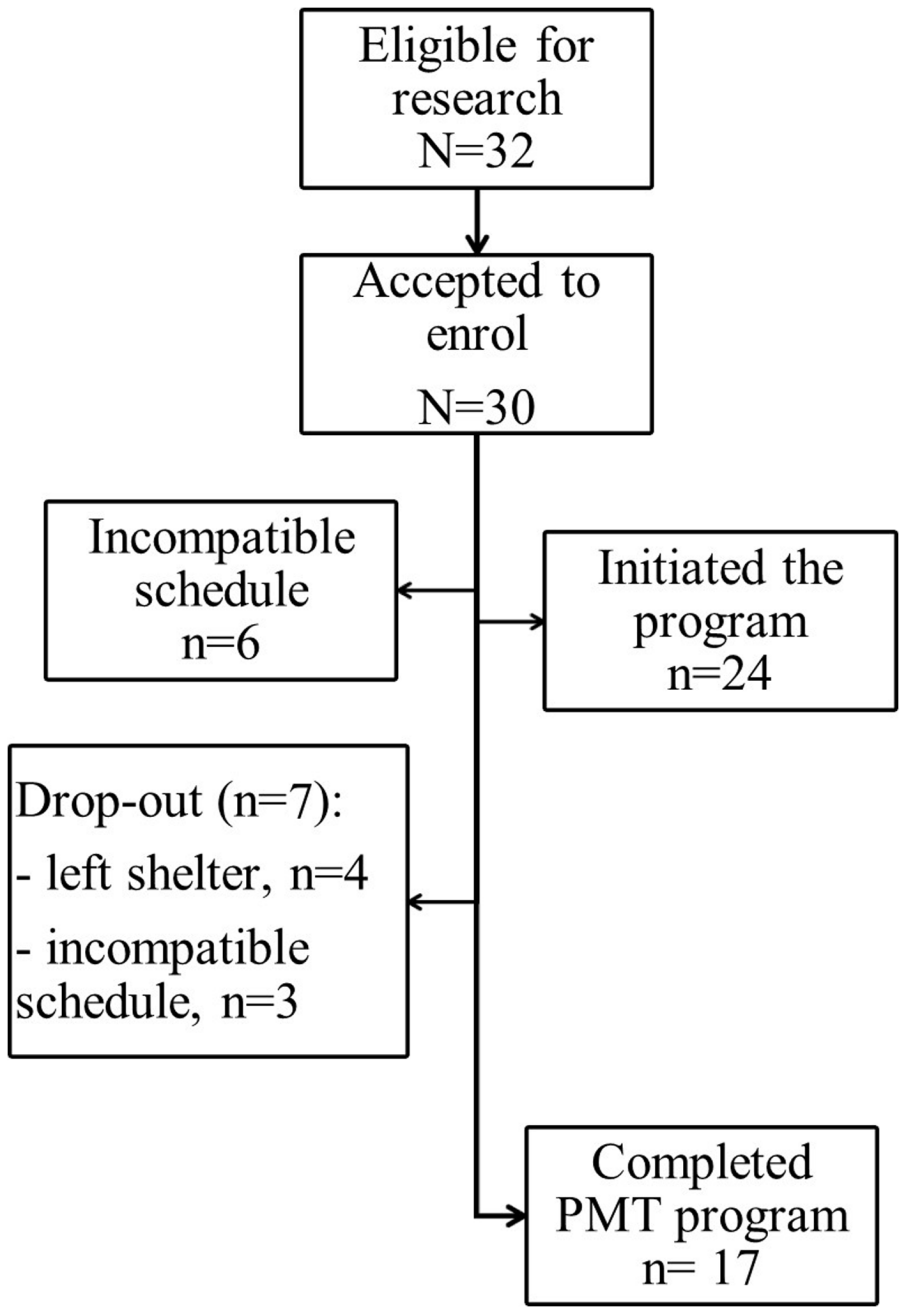
Flow diagram of recruitment and participation.

A non-random within-group repeated measures design was used to evaluate the effects and feasibility of FOM on female victims of IPV living in shelter homes. Participants were tested at week 1 (T1) and week 5(T2) before the intervention, to monitoring of the control period. Participants were again tested at week 13, after the 8-week intervention (T3). The study was previously approved by the University ethics committee and conducted in accordance with the Declaration of Helsinki (General Assembly of the World Medical Association, 2014). From June 2021 to November 2022, this intervention research was proposed to the managing entities of three Portuguese shelters for victims of IPV. Upon agreement, women victims who were currently living in each shelter were invited by the managing entities to attend a brief presentation session in the shelter. The study procedures (assessments needed) and characteristics of the intervention (length, frequency of the sessions, therapist and types of activities) were presented by the therapist of FOM to the women who attended this session and who were considered eligible to participate in the study. The inclusion criteria were being 18 years or older and having suffered IPV in the first person. Ninety-four percent of the women showed an interest in participating (*N* = 30), as represented in Figure 1.

### Participants

Thirty women accepted to enroll in this study. Due to incompatible schedules (considering work and mothering tasks), 6 women could not engage in the program. Of the 24 initial participants, seventeen (mean age 42.8 years, sd = 11.1; range 21–64) completed the program. Those reported that they had suffered physical (71%), psychological (94%), and sexual (59%) violence for a mean duration of 16 years and 3 months (sd = 16.5 years; range 2–48 years) and were free from violence for a mean of 6 months (sd = 0.53 years; range: 1–18 months). In Table 1 we can see that, at baseline, 82% (*n* = 14) of the participants were unemployed or retired, and 71% (*n* = 12) were living in the shelter with one or more children. In the sociodemographic survey, the most reported symptoms were sleep problems (65%), chronic pain (41%), and anxiety attacks (29%).

**Table 1:** Descriptive statistics of demographic and health information.

Sociodemographic variables	*N* = 17		
	*N* (%)	Mean (SD)	Range
Age (years)		42.8 (11.1)	21–64
Body Mass Index (kg/m^2^)		27.7 (5.1)	18.7–37.6
Types of violence			
Psychological only	4 (24)		
Psychological and Physical	3 (18)		
Psychological, Physical and Sexual	10 (59)		
Duration of violence (years)		16.3 (16.5)	2.0–48.0
Time since violence ended (years)		2.4 (6.1)	0.1–30.0
Health			
Sleep problems	11 (65)		
Anxiety attacks	5 (29)		
Migraines	4 (24)		
Memory difficulties	2 (12)		
Chronic pain	7 (41)		
Gastrointestinal problems	4 (24)		
Hypertension	2 (12)		
Medical diagnosis			
Posttraumatic stress disorder	1 (6)		
Anxiety disorder	5 (29)		
Depression disorder	9 (53)		
Behavior			
Self-injury	5 (29)		
Suicidal ideation	9 (53)		

M, mean; SD, standard deviation.

### Procedures

After signing the informed consent form, the baseline assessment session was scheduled. Participants completed a sociodemographic survey about general health symptoms, medical diagnosis of posttraumatic stress disorder, anxiety and depression, use of psychiatric medication, leisure activities or therapeutic practices, and the types of violence they have suffered, for how long, and since when they were free from the violent relationship. Participants completed the PTSD Checklist (Marcelino and Gonçalves, 2012), the Hospital Anxiety and Depression Scale (HADS; Pais-Ribeiro et al., 2007), Scale of Body Connection (SBC, Neves et al., 2017), and World Health Organization Quality of Life checklist (WHOQoL-bref; Vaz Serra et al., 2006). Assessment sessions took about 60 min, and occurred at baseline (T1), after 4 weeks of control time (pre-intervention, T2), and after 8 weeks of intervention (post-intervention, T3).

### Instruments

#### Posttraumatic stress disorder checklist

Posttraumatic Stress Disorder symptoms in the last 2 months were evaluated using the PTSD Checklist–civilian version (PCL; Weathers et al., 1994). This self-report questionnaire can differentiate the three PTSD clusters from the DSM-IV medical diagnostic manual: experiencing, avoidance, and hypervigilance. A Likert scale, from zero (nothing) to five (extremely) was used to score the frequency of each symptom. The sum of the scores for each cluster was analyzed. The Portuguese version includes 17 items and has good psychometric properties (Cronbach’s alpha = 0.94; Marcelino and Gonçalves, 2012).

#### Hospital anxiety and depression scale

Hospital Anxiety and Depression Scale (HADS; Zigmond and Snaith, 1983) enables health professionals to assess anxiety and depression levels at a brief and objective way (Herrmann, 1997). It is a self-report questionnaire with 14 items, seven assessing anxiety and seven assessing depression symptoms. Each item is rated in a 0–3 scale and classifies the symptom as feeling equal to times before or a lot worse than before. Scores higher than 7 in each subscale, indicate clinically relevant levels of anxiety or depression, and results can be analyzed through the sum of the items of each subscale, ranging from 0 to 21. Snaith (2003) suggests scores of 0–7 to be considered “normal,” between 8 and 10 being suggestive of the presence of the state of anxiety or depression, and above 11 to be indicative of the presence of that state. The most recent study found the Portuguese version to have good internal consistency (Cronbach’s alpha_anxiety scale = 0.76; Cronbach’s alpha_depression scale = 0.81; Pais-Ribeiro et al., 2007).

#### Scale of body connection

Bodily Dissociation (the sense of separation from the body) was assessed through the Scale of Body Connection (SBC; Price and Thompson, 2007), a self-report Likert scale that measures body awareness and bodily dissociation. Mean scores higher than 2 suggest a significant presence of bodily dissociation symptoms. The adaptation from Neves et al. (2017) confirmed the reliability and validity of this scale for the Portuguese population (Cronbach’s alpha = 0.73).

#### World health organization quality of life checklist

The WHOQoL is a self-administered Likert scale that allows the assessment of the perceived quality of life in four domains: Physical health (domain 1; e.g. dependence on medical aids, mobility, pain, work capacity), Psychological health (domain 2; e.g. negative and positive feelings, self-esteem, body image, personal beliefs, learning and memory), Social relationships (domain 3; e.g. personal relationships, social support, sexual activity) and Environment (domain 4; e.g. freedom, physical safety, accessibility to health and social care, opportunities for acquiring new information and to participate in recreative activities, transport). The Portuguese version of the WHOQoL-bref includes 26 items and shows good reliability and validity scores, except for domain 3 (Cronbach’s alphas ranging from 0.64 for domain 3 and 0.87 for domain 1; Vaz Serra et al., 2006), which was thus removed from analysis in this study (Tavakol and Dennick, 2011).

### The Feel-Own-Move psychomotor therapy program

#### Psychophysiology of trauma

Neural circuits involved in interoception (integration and processing of visceral bodily sensations) and emotional regulation, as the insula, the amygdala and the prefrontal cortex seem to be disrupted as a result of trauma experiences (Bruce et al., 2013; Nicholson et al., 2015). In fact, not only depression and anxiety, but also PTSD reflects impairments in emotional and arousal regulation (Shepherd and Wild, 2014). Acknowledging those impairments and their chronic dysregulation among victims of trauma, Payne et al. (2015) proposed a therapeutic somatic restoration of the Core Response Network (CRN), a brain network connecting the limbic, autonomic, motor and arousal systems. The authors state that people with PTSD are stuck with a dysregulated CRN, translating into hypo or hyper-arousal chronic states (Levine, 2010; Payne et al., 2015). Further research has also found that for victims of trauma, chronic stress “inhibits the effectiveness of the stress response and induces desensitization” (Van der Kolk, 2022, p. 15). With this in mind, (i) exercise- and movement-based interventions aiming to recover access to internal bodily sensations, and (ii) breathing and meditation techniques aiming to promote emotional regulation abilities in both a psychophysiological and behavioral axis, have been studied (Caldwell and Victoria, 2011; Rosenbaum et al., 2015; Taylor et al., 2020; Björkman and Ekblom, 2022).

#### The Feel-Own-Move rationale

Based on the exposed literature, FOM was developed to allow victims of IPV to regain awareness of bodily sensations, to integrate sensations and emotions into the senses of body ownership and agency, and to train abilities of arousal regulation. Through movement, expression, breathing, and relaxation techniques, the aim of each session was twofold. On the one hand, to progressively promote and deepen a non-judgmental awareness of bodily sensations and of sensation-emotion relationships, strengthening the mind–body connection. On the other hand, the second aim was to increase self-regulation, as a path to weaken mental health symptoms, and indirectly increase the quality of life. The program combined individual sessions and group sessions.

The individual sessions had an approximate length of 40 min and allowed for personalized attention to the bodily sensations, representations, and expressions of each participant. This individualization offered a specific therapeutic space and time, for a deepened exploration and integration of each insight. In addition, group sessions (4–6 participants), with approximately 60 min each, allowed for an empathetic expression of each participant, offering a meeting of different ways of embodied being. The encounters promoted by group sessions aimed for a widening of images, sensations, and representations into the therapeutic process of each participant.

Overall, both individual and group sessions included three sequential moments: an initial warming-up activity, a second moment with body awareness and grounding activities and a final relaxation moment.

#### Warming-up

The activation of proprioceptive (muscular) and interoceptive (visceral) sensations in the initial moments of each session was accomplished through aerobic exercises and strength training (Powers et al., 2015; Rosenbaum et al., 2015; Björkman and Ekblom, 2022). The benefits of exercise, especially aerobic exercise, in the treatment of PTSD-related symptoms is hypothesized as being due to the elevation of brain-derived neurotrophic factor, related with synaptic plasticity (Powers et al., 2015). In FOM, the exacerbation of neutral bodily sensations through exercise has the aim of facilitating awareness in cases of bodily dissociation and hypo-arousal states (Machorrinho et al., 2022). Distinctively, these activities are frequently accompanied by bodily metaphors and movement imagery to increase connection to the body and the feeling of empowerment. Importantly, exercise has to be proposed with an attuned approach, i.e., process-oriented, highlighting safety sensations and a joyful experience (Calogero et al., 2019). This attunement with exercise and a careful adjustment to participant’s capacities, ensures for optimal effectiveness, promotes motivation and reduces dropouts (Louková et al., 2015; Calogero et al., 2019; Van de Kamp and Hoven, 2019). This exacerbation of bodily sensations serves as a starting point to the reclaiming of ownership and agency toward the body, enhanced in the following moment of the session (MacLaren, 2016).

#### Body awareness and grounding

It is important for patients with dissociative symptoms to also improve sensory awareness in a slow, integrative and non-judgmental approach (Ogden et al., 2006; Van der Kolk, 2015). The integration of bodily sensations and of the body–mind connection is frequently enhanced in yoga and grounding techniques as a path to stabilization and peaceful reconnection to the body (Brand et al., 2012; O’Shea Brown, 2021). In FOM, the therapist facilitates the transition to a second moment of slow movements, using therapeutic touch, imagery, or guided sensations (saying, for example, “Focus on the weight of the body against the wall,” or asking “Where in your body do you feel strength/ resistance/ movement/ stillness?”) as mediators. These mediators aim to reinforce the body–mind connection and the feelings of body ownership and agency (Kirmayer and Gómez-Carrillo, 2019). Body schema activities are also promoted in these moments to surpass the segmentation of the body and promote its wholeness (Louková et al., 2015; Marmeleira et al., 2023).

#### Relaxation

The regulation of arousal seems to be a crucial element to account for when intervening in trauma-related disorders (Van de Kamp and Hoven, 2019). Relaxation and breathing techniques are frequently delivered with the intent of reducing patients’ excessive physiological arousal and promoting emotional regulation skills. In FOM, sessions end with physiologic-driven relaxation techniques such as Jacobson’s progressive muscle relaxation and active-passive relaxation proposed by Wintrebert (Guiose, 2003). Progressive muscle relaxation is used in the initial phase, as a present-focused and active relaxation, which can be easily translated to quick practices for patients’ use in daily life (Hazlett-Stevens and Fruzzetti, 2021). Once the participants can autonomously endorse general progressive relaxation, the active-passive relaxation method is implemented. In the last sessions of FOM, participants are invited to train mindfulness meditation techniques to address daily arousal regulation needs.

#### Implementation

The program consisted of an 8-week Psychomotor Therapy (PmT) with 24 three-weekly sessions, combining 16 individual sessions with 8 group sessions. The intervention was delivered by two therapists with more than 12 years of clinical experience in body–mind-oriented interventions. The program was developed, structured, and conceptualized by one of them (as part of the research team), who also holds a bachelor’s and master’s degree in psychomotricity. A manual guide and protocol of implementation for the FOM program were previously developed and trained by the therapists to ensure maintenance of the FOM’s purpose and delivery. Both therapists had permanent intervision and mutual monitoring of the process, struggles experienced, and qualitative achievements. Both therapists also had weekly supervision with a third therapist from the research team that developed FOM, with more than 20 years of experience in body-psychotherapeutic practice and supervision.

### Feasibility and acceptability

The reach of the program and adherence of the participants was assessed to examine its feasibility. To examine acceptability and satisfaction with the PmT intervention, a 9-item survey developed by the authors of this study was administrated to the participants who completed the program. Following recommendations of Bowen and colleagues (Bowen et al., 2009), each item was classified on a Likert scale, between zero (nothing) and four (extremely), and covered topics related to perceived personal impact, sense of trust and respect and comfort regarding the sessions, the therapist, and the assessments.

### Data analysis

A descriptive analysis of sociodemographic and health variables was performed. The normality of data was checked through the Shapiro–Wilk test. A one-way repeated measures ANOVA was used to examine within-group changes between T1 and T2, and T2 and T3. Significance levels were adjusted using the Bonferroni correction, considering significance if *p* < 0.05. Mean and standard deviations are reported. Effect sizes are provided as partial eta-squared (η_p_^2^) and interpreted as: 0.01–0.06, small effect, 0.06–0.14, medium effect, and ≥ 0.14, large effect (Cohen, 1988). Results of non-parametric variables are presented as median and interquartile range (IQR). Friedman tests were carried out to examine changes in non-parametric variables, using *post hoc* pairwise comparisons (Wilcoxon Signed-Rank test) and a Bonferroni adjustment was applied. Significance levels were considered at *p* < 0.017. Effect sizes were calculated using Kendall’s W Value, and interpreted as <0.3, small effect, 0.3–0.5, moderate effect, and > 0.5, large effect (Tomczak and Tomczak, 2014). The delta value (Δ%) of proportional change between each moment (T1, T2, and T3) was calculated using the formula: Δ% = [(momentY – (momentY−1))/(momentY-1)] x 100.

Statistical analyzes were performed, using SPSS version 24.0 software (IBM Corp, 2017).

## Results

Overall, the results suggested good feasibility of the program. Regarding reach, it was measured as the rate of women who accepted to participate in the study, from all the ones who were invited to. Thirty out of 32 agreed to participate (94%). The two women who declined had just arrived at the shelter (2 or 3 days before the invitation) and claimed not to be prepared to initiate a therapeutic process yet. Due to schedule incompatibilities, 6 women were not able to integrate the program. In those cases, women had intense and rotating schedules, added to house and mothering chores. Twenty-four women initiated the program, and seven drop-out, representing a retention rate of 71%. Considering adherence, among participants who completed the program, they attended 86% of the individual sessions and 75% of the group sessions. Results showed strong acceptability and satisfaction with the program, as detailed in Table 2 (all positive questions ≥3.5).

**Table 2:** Acceptability and satisfaction with FOM.

Questions	Mean (SD)	Range
Do you feel satisfied with the program you have participated in?	3.9	3–4
Do you feel yourself different from before initiating the program?	3.5	2–4
Do you feel that this program brought positive things to you?	0.8	2–4
Were the activities of the sessions interesting?	3.7	2–4
Did you felt respected in the sessions?	3.9	3–4
Do you feel that this program brought negative things to you?	0.0	0
Did you felt that you could trust in the therapist?	4.0	4
Did you felt yourself safe during the sessions?	3.9	3–4
Did you felt bothered/ disturbed with the assessments?	1.2	0–4

Values ranging from zero (nothing) to four (extremely).

Results show a significant decrease in levels of bodily dissociation over time [*F*(2) = 4.517, *p* = 0.029, η_p_^2^ = 0.376]. *Post hoc* analysis with Bonferroni adjustment revealed that bodily dissociation decreased from pre (*M* = 2.3, SD = 0.8) to post-intervention (*M* = 2.0, SD = 0.7) (∆% = 12,4%). Scores of the environmental quality of life showed a non-significant increase between assessments, although a large effect size was found [*F*(2) = 1.543, *p* = 0.246, η_p_^2^ = 0.171]. The results of all dependent variables are shown in Table 3.

**Table 3:** Scores on dependent variables at baseline (T1), pre-intervention (T2) and post-intervention (T3).

	Baseline	Pre-intervention	Post-intervention	*p*	∆%	Effect size
PTSD						
Reexperiencing^a^	12.6 (6.0)	12.2 (6.0)	11.0 (13.0)	0.808		
Hyper-vigilance^b^	12.9 (5.2)	12.2 (5.2)	12.3 (5.3)	0.749		
Avoidance^a^	15.0 (8.0)	14.0 (15.0)	14.0 (11.5)	0.345		
Anxiety^b^	9.9 (4.1)	9.3 (5.1)	9.0 (5.5)	0.762		
Depression^a^	7.0 (5.0)	6.0 (5.0)	7.0 (5.5)	0.405		
Bodily dissociation^b^	2.3 (0.8)	2.3 (0.8)	2.0 (0.7)	0.029	T3 < T2 (12.4%)	.376^d^
Quality of life						
Physical^a^	3.2 (0.9)	3.3 (1.0)	3.1 (0.8)	0.939		
Psychological^b^	3.7 (0.6)	3.5 (0.7)	3.6 (0.6)	0.467		
Environment^b^	3.1 (0.5)	3.0 (0.5)	3.3 (0.5)	0.246	T3 > T2 (10.0%)	.171^d^

PTSD, Posttraumatic Stress Disorder; ^a^data reported as median (interquartile range); ^b^data reported as mean (standard deviation); ∆%, proportional change; ^c^effect size reported as Kendall’sW value; ^d^effect size reported as partial eta-squared, η_p_^2^.

## Discussion

The study of the associations between mental health and embodiment-related variables among victims of IPV has gained paramount importance (Machorrinho et al., 2021b, 2022). Although there is increasing research on therapeutic and preventive interventions for victims of trauma, highlighting body–mind interactions and their influence on victims’ health, trauma recovery, and quality of life, the necessary adaptations for victims of IPV were yet scarcely attended (Van der Kolk, 2015; Marmeleira et al., 2023). The aim of the present study was to examine the effects of a Psychomotor Therapy “Feel-Own-Move” (FOM) program delivered in shelter homes for victims of IPV. The FOM program was shown to be effective at decreasing the values of bodily dissociation in victims, also with a suggested effect on increasing quality of life.

The recruitment and retention of participants in IPV-related research have been a longstanding concern (Dutton et al., 2003). In this study, the attractiveness of the body–mind program, the schedules flexibility and adaptation to participants’ possibilities, the close positive recommendation of the shelters’ managing entities, and the empathic attitude of the PmT therapist, might have been important factors to the excellent reach of the program (94% of women accepted to enroll in the study; Dutton et al., 2003). Nevertheless, 7 of the 24 participants (29%) did not complete the program, mostly because they have left the shelter to rebuild their lives in another city or to move to another shelter with more adequate social-economic support. Although this is a positive retention rate compared with other IPV research (Hansen et al., 2014; Arroyo et al., 2017), it is important to note that the study design here implemented may have influenced the retention rate of the program, since it compelled participants to a 4-week control period before initiating the intervention. Without this control period, inherent to the research, we can hypothesize that more participants would be able to complete the program, since its short duration is in accordance with current recommendations of interventions to be delivered in shelter homes (Arroyo et al., 2017). Moreover, to surpass the schedule constraints pointed out by 13% of the initial participants who dropped out, we suggest that in the future the FOM should provide a set of videotaped sessions that participants can easily watch and perform individually at any time. Although these would imply close supervision from the psychomotor therapist, we believe that it could be advantageous for the feasibility of the FOM program.

IPV is equally prevalent across all age groups, and shelter homes welcome adult women with no age grouping. Thus, as expected, our sample included women in a wide age range. More than half (59%) have suffered the three types of violence (sexual, physical and psychological), which is known to increase the repercussions of trauma on mental health and quality of life (Campbel, 2002). At baseline, participants mostly reported symptoms of chronic pain, difficulty falling asleep and staying asleep, and anxiety attacks. Adding to this, self-reported measures at baseline revealed clinically relevant levels of anxiety, bodily dissociation and PTSD (avoidance cluster), but only normal to light levels of depressive symptoms. Conversely, depressive disorder was the most medically diagnosed in this sample (*n* = 9; 53%), whereas only 5 participants (29%) had a previous diagnosis of anxiety disorder, and only one (6%) of PTSD. This finding suggests an underdiagnosis of trauma-related mental health problems, possibly due to women not having any medical/ psychological check-ups after they have exposed their IPV victimization. Suicidal ideation and self-injury behaviors, which are commonly an expression of depressive feelings and of a disconnection from the body, were highly reported in this sample (You et al., 2012; Hielscher et al., 2019; Polskaya and Melnikova, 2020). It is also noteworthy the high levels of body mass index (BMI), which were recently suggested as an indicator of weaker self-care behaviors and health-promoting attitudes in IPV victims, mediating the associations between IPV and physical health problems (Weaver and Resnick, 2004; Machorrinho et al., 2022).

Prior to intervention, a control period allowed for important monitoring of each variable evolution, since the shelter home, as a preventive and supportive strategy for victims, can have a positive impact on their well-being and mental health (Yakubovich et al., 2022). However, none of the variables have shown significant differences between baseline and pre-intervention assessments, which highlights the lack of effective short-term therapeutic interventions at these shelters.

The FOM program allowed women to reconnect with their bodies in a trustworthy relational atmosphere. Along with the relevant decreases in bodily dissociation levels, participants ended up being extremely satisfied with the program, reporting feelings of safety, respect and trust toward the setting and the therapist. These results corroborate research that suggests both physical and relational safety as a primary step for effective intervention with trauma clients (Baylin and Winnette, 2016).

While bodily dissociation is importantly related with poorer quality of life and physical and mental health, its improvement through therapeutic approaches is yet scarcely studied (Price, 2007; Price et al., 2012; Cheng et al., 2022). Bodily dissociation acts through a disregard of internal experience, an avoidance of sensations and emotions, thus interfering with one’s monitoring of health and self-care behaviors (Price and Thompson, 2007). The avoidance or dissociation from bodily experience is part of the concept of psychophysical awareness, linked to the internal processes necessary for self-regulation and self-knowledge (Price and Thompson, 2007). Bodily dissociation thus causes significant impairments in daily life and impoverishes the possible outcomes of psychological therapeutic processes (Price et al., 2017).

While various body–mind interventions have shown to be effective at increasing body awareness (the awareness of body sensations, also part of psychophysical awareness), impacting bodily dissociation has shown to be more difficult, especially when considering short-term interventions (Classen et al., 2021; Cheng et al., 2022). A possible explanation mechanism for the positive effect of this Psychomotor therapy program on bodily dissociation is the inclusion of cardiovascular and strength activities with an attuned approach (Louková et al., 2015; Calogero et al., 2019; Marmeleira et al., 2023). These activities were delivered with joyful, safe and process-oriented instruction, again embedded in an empathic and encompassing therapeutic setting. By (i) increasing biological sensations of movement, rhythm, and vitality, while (ii) emphasizing the connections between sensations and emotions, and (iii) delivering words of agency toward the body, these activities aimed to facilitate awareness and reconnect female victims of IPV with their living bodies, allowing them to regain their sense of body ownership (Machorrinho et al., 2022; Marmeleira et al., 2023). This mechanism might also explain the large effect size of the intervention on the environmental domain of quality of life. This domain refers to senses of safety, mobility, autonomy and opportunity to participate in leisure activities, which corroborates the rationale of the Psychomotor therapy intervention where some of the short-term objectives are to activate movement, to regain pleasure in physical activity, and to increase vitality and strength (Marmeleira et al., 2023). On the other hand, research has shown benefits of exercise on symptoms of PTSD, anxiety and depression, especially for aerobic exercise, yoga practices and general physical activity (Cabral et al., 2011; Hallgren et al., 2016; Frederiksen et al., 2021). Rosenbaum et al. (2015) specifically found that adding exercise augmentation through motivational tools to decrease sedentary behavior, and resistance training, is more effective at reducing PTSD than treatment as usual only. Through a systematic review with meta-analysis, Björkman and Ekblom (2022) corroborated the overall benefits of adding exercise to the usual treatment of PTSD. The hypothesis of why FOM did not had similar significant positive results on PTSD is twofold. First, because, although FOM included an initial exercise moment with the aim of augmenting interoceptive and proprioceptive sensations, this was not the main or only goal of FOM, justified by the inclusion of two other important moments in each session. Second, the majority of the interventions revised by Björkman and Ekblom (2022) were implemented for at least 12 weeks, with 2–3 sessions per week. Due to the retention concerns inherent to the IPV shelter context, FOM had a shortened duration of 8 weeks, which can possibly have a limiting effect on its results, specifically on effectively reducing PTSD symptoms.

Overall, results suggest that FOM has an impact on reconnecting women victims of IPV with their bodies, diminishing their bodily dissociation and promoting their willingness to move more and autonomously engage in activities. This program had, however, no significant effects on reducing anxiety, reexperiencing, or hyper-vigilance, nor on their perceived quality of life in terms of physical, social, or psychological healthcare. It is thus important to notice that many of those women were on a waiting list for physical therapy, reconstruction surgery, or general medical and psychological assistance related to serious sequelae from the violent assaults. Additionally, as time goes by in shelter homes, each woman is under increased pressure to leave, find a job, and provide children enough social and economic autonomy, which can increase their feelings of anxiety and fear of being somehow a victim again.

### Limitations

One factor that limits the generalizability of the results of FOM is the small sample size. Efforts were made to attend to different shelters and balance participants’ heterogeneity in order to enrich the depth of results. However, the sample size was negatively impacted due to the loss of participants from the shelters. This loss was due, not only by women leaving the shelter or finding occupying jobs, but also by the therapeutic-groups design, that required the ending of one program before initiating another one, which delayed the inclusion of new participants in the study, possibly extending its costs. One other factor that might have limited the impact of FOM, was the poor conditions of the shelter to effectively implement the relaxation moments during sessions. In general, the small, cold and loud rooms made available for therapy were not adequate to practice relaxation and hindered the therapist’s efforts to counterbalance such limitations. Also, some sessions’ length had to be shortened due to women not having no one in the house to take care of the children. This can reveal an important concern when reflecting on the feasibility of FOM’s implementation. Future FOM implementations might benefit from having a contingency plan with appropriate conditions to the relaxation activities, and plan for parallel activities for the children.

IPV is a complex problem that requires extensive research on various psychological, physical and social dimensions. Acknowledging the intertwining of most of those dimensions, it would be important to account for confounding variables in this research. This was not possible to perform in the current study due to the non-normal distribution of some of the dependent variables, which prevented us from supporting a reliable covariance statistical analysis. In this regard, the account for confounding variables is recommended for future studies. Also, a multidimensional assessment of variables that can impact trauma experience and adherence to support is also recommended, as it is the example of attachment security.

We further encourage future research to examine the evolution of FOM’s results on follow-up assessments. Additionally, we suggest exploring the effectiveness of FOM on reoccurrence rates, acknowledging that bodily dissociation was recently hypothesized as playing an important role in revictimization numbers (Zamir et al., 2018). Considering the positive effects found in the present study, it could be important to adapt FOM to be extended to other levels of IPV prevention, such as primary support centers and healthcare settings.

In conclusion, FOM, an 8-week Psychomotor therapy program seems to be a feasible and highly accepted therapeutic intervention for female victims of IPV living in shelters. Most importantly, this program showed to be effective in reducing bodily dissociation among participants, which is suggested to prospectively contribute to their mental health and quality of life (Machorrinho et al., 2021b).

## Data availability statement

The raw data supporting the conclusions of this article will be made available by the authors, without undue reservation.

## Ethics statement

The studies involving human participants were reviewed and approved by Ethics Committee of the University of Évora. The patients/participants provided their written informed consent to participate in this study.

## Author contributions

JM, GS, GV, and JMar contributed to the design and conception of this study. JM and GS cautiously designed the intervention program, which was critically revised by GV and JMar. JM collected data, which was analyzed by JM and JMar. JM wrote the first draft of the manuscript. GS, GV, and JMar revised the final manuscript. All authors contributed to manuscript revision, read, and approved the submitted version.

## Funding

This research was funded by Fundação para a Ciência e Tecnologia, IP National support through Comprehensive Health Research Centre, CHRC (UI/BD/150985/2021). Also, this work is funded by national funds through the Foundation for Science and Technology, under the project UIDP/04923/2020.

## Conflict of interest

The authors declare that the research was conducted in the absence of any commercial or financial relationships that could be construed as a potential conflict of interest.

## Publisher’s note

All claims expressed in this article are solely those of the authors and do not necessarily represent those of their affiliated organizations, or those of the publisher, the editors and the reviewers. Any product that may be evaluated in this article, or claim that may be made by its manufacturer, is not guaranteed or endorsed by the publisher.

## References

[ref1] Andersen T. E. Lahav Y. Ellegaard H. Manniche C. (2017). A randomized controlled trial of brief somatic experiencing for chronic low back pain and comorbid post-traumatic stress disorder symptoms. Eur. J. Psychotraumatol. 8:1331108. doi: 10.1080/20008198.2017.1331108, PMID: 28680540 PMC5489867

[ref2] Arroyo K. Lundahl B. Butters R. Vanderloo M. Wood D. S. (2017). Short-term interventions for survivors of intimate partner violence: a systematic review and meta-analysis. Trauma Violence Abuse 18, 155–171. doi: 10.1177/1524838015602736, PMID: 26335794

[ref3] Ataria Y.. (2018). Body disownership in complex posttraumatic stress disorder. New York, US: Palgrave Macmillan.

[ref4] Baylin J. Winnette P.. (2016). Working with traumatic memories to heal adults with unresolved childhood trauma: Neuroscience, attachment theory and Pesso Boyden system psychomotor psychotherapy. London: Jessica Kingsley Publishers.

[ref5] Bieleveldt S.. (2019). *Psychomotor therapy and recovery of intimacy: the importance of raising body awareness and to increase a positive experience of intimacy and sexuality*. ESTSS Symposium, pp. 2–4.

[ref6] Björkman F. Ekblom Ö. (2022). Physical exercise as treatment for PTSD: a systematic review and meta-analysis. Mil. Med. 187, e1103–e1113. doi: 10.1093/milmed/usab497, PMID: 34850063

[ref7] Bowen D. J. Kreuter M. Spring B. Cofta-Woerpel L. Linnan L.. (2009). How we design feasibility studies. Am. J. Prev. Med. 36, 452–457. doi: 10.1016/j.amepre.2009.02.002, PMID: 19362699 PMC2859314

[ref8] Brand B. L. Myrick A. C. Loewenstein R. J. Classen C. C. Lanius R. McNary S. W.. (2012). A survey of practices and recommended treatment interventions among expert therapists treating patients with dissociative identity disorder and dissociative disorder not otherwise specifed. Psychol. Trauma Theory Res. Pract. Policy 4:490500, 490–500. doi: 10.1037/a0026487

[ref9] Bruce S. E. Buchholz K. R. Brown W. J. Yan L. Durbin A. Sheline Y. I. (2013). Altered emotional interference processing in the amygdala and insula in women with post-traumatic stress disorder. Neuroimage Clin. 2, 43–49. doi: 10.1016/j.nicl.2012.11.003, PMID: 24179757 PMC3777837

[ref10] Cabral P. Meyer H. B. Ames D. (2011). Effectiveness of yoga therapy as a complementary treatment for major psychiatric disorders: a meta-analysis. Prim. Care Companion CNS Disord. 13:1068. doi: 10.4088/PCC.10r01068, PMID: 22132353 PMC3219516

[ref11] Caldwell C. Victoria H. K. (2011). Breathwork in body psychotherapy: towards a more unified theory and practice. Body Mov. Dance Psychother. 6, 89–101. doi: 10.1080/17432979.2011.574505

[ref12] Calogero R. Tylka T. McGilley B. Pedrotty-Stump K. (2019). “Attunement with exercise (AWE)” in Handbook of positive body image and embodiment: Constructs, protective factors, and interventions. ed. Tylka T. L. (New York: Oxford Academic)

[ref13] Campbel J. C. (2002). Health consequences of intimate partner violence. Lancet 359, 1331–1336. doi: 10.1016/S0140-6736(02)08336-8 11965295

[ref14] Cheng S. C. Thompson E. A. Price C. J. (2022). The scale of body connection: a multisample study to examine sensitivity to change among mind–body and bodywork interventions. J. Integr. Complement. Med. 28, 600–606. doi: 10.1089/jicm.2021.0397 35452263

[ref15] Classen C. C. Hughes L. Clark C. Hill M. B. Woods P. Beckett B. (2021). A pilot RCT of a body-oriented group therapy for complex trauma survivors: an adaptation of sensorimotor psychotherapy. J. Trauma Dissoc. 22, 52–68. doi: 10.1080/15299732.2020.1760173, PMID: 32419670

[ref16] Cohen J. (1988). Statistical power analysis for the behavioral sciences, 2nd Edn.. Hillsdale: Lawrence Erlbaum.

[ref17] Coker A. L. (2004). Primary prevention of intimate partner violence for Women’s health. J. Interpers. Violence 19, 1324–1334. doi: 10.1177/0886260504269686 15534334

[ref18] Costa D. Hatzidimitriadou E. Ioannidi-Kapolou E. Lindert J. Soares J. Sundin Ö.. (2015). Intimate partner violence and health-related quality of life in European men and women: findings from the DOVE study. Qual. Life Res. 24, 463–471. doi: 10.1007/s11136-014-0766-9, PMID: 25063083

[ref19] Dieterich-Hartwell R. (2017). Dance/movement therapy in the treatment of post traumatic stress: a reference model. Arts Psychother. 54, 38–46. doi: 10.1016/j.aip.2017.02.010

[ref20] Dutton M. A. Holtzworth-Munroe A. Jouriles E. McDonald R. Krishnan S. McFarlane J.. (2003). Recruitment and retention in intimate partner violence research. Rockville, US: US Department of Justice.

[ref21] Eckhardt C. I. Murphy C. M. Whitaker D. J. Sprunger J. Dykstra R. Woodard K. (2013). The effectiveness of intervention programs for perpetrators and victims of intimate partner violence. Partn. Abus. 4, 196–231. doi: 10.1891/1946-6560.4.2.196

[ref22] Emck C. Scheffers M. (2019). “Psychomotor interventions for mental health: an introduction” in Psychomotor interventions for mental health–adults. eds. De Lange J. Glas O. Van Busschbach J. Emck C. Scheewe T. (Los Angeles, CA: Boom), 162–179.

[ref23] European Forum of Psychomotricity. (2012). Psychomotrician professional competences in Europe. Ljubljana: European Forum of Psychomotricity.

[ref24] Fernandes J. Veiga G. Gutierres F. P. (2022). Psicomotricidade e paradigma da complexidade. Psicol. Saúde Debate 8, 363–377. doi: 10.22289/2446-922X.V8N1A21

[ref25] Frederiksen K. P. Stavestrand S. H. Venemyr S. K. Sirevag K. Hovland A. (2021). Physical exercise as an add-on treatment to cognitive behavioural therapy for anxiety: a systematic review. Behav. Cogn. Psychother. 49, 626–640. doi: 10.1017/S1352465821000126, PMID: 33678210

[ref26] General Assembly of the World Medical Association (2014). World medical association declaration of Helsinki: ethical principles for medical research involving human subjects. J. Am. Coll. Dent. 81, 14–18. PMID: 25951678

[ref27] Guiose M. (2003). “Fondements théoriques et techniques de la relaxation,” in Rapport, medicine faculty de. ed. Faculté de médecine Pierre et Marie Curie (Paris: Paris-VI University), 21–23.

[ref28] Hallgren M. Herring M. P. Owen N. Dunstan D. Ekblom O. Helgadottir B.. (2016). Exercise, physical activity, and sedentary behavior in the treatment of depression: broadening the scientific perspectives and clinical opportunities. Front. Psych. 7:36. doi: 10.3389/fpsyt.2016.00036 PMC4786540 27014101

[ref29] Hansen N. B. Eriksen S. B. Elklit A. (2014). Effects of an intervention program for female victims of intimate partner violence on psychological symptoms and perceived social support. Eur. J. Psychotraumatol. 5:24797. doi: 10.3402/ejpt.v5.24797, PMID: 25279107 PMC4163755

[ref30] Hazlett-Stevens H. Fruzzetti A. E. (2021). “Regulation of physiological arousal and emotion” in Handbook of cognitive behavioral therapy: Overview and approaches. ed. Wenzel A. (Washington, DC: American Psychological Association), 349–383.

[ref31] Herrmann C. (1997). International experiences with the hospital anxiety and depression scale-a review of validation data and clinical results. J. Psychosom. Res. 42, 17–41. doi: 10.1016/S0022-3999(96)00216-4, PMID: 9055211

[ref32] Hielscher E. Whitford T. J. Scott J. G. Zopf R. (2019). When the body is the target—representations of one’s own body and bodily sensations in self-harm: a systematic review. Neurosci. Biobehav. Rev. 101, 85–112. doi: 10.1016/j.neubiorev.2019.03.007, PMID: 30878499

[ref33] IBM Corp. (2017). IBM SPSS statistics for windows, version 24.0. Armonk, NY: IBM Corp.

[ref34] Kelly U. (2010). Intimate partner violence, physical health, posttraumatic stress disorder, depression, and quality of life in Latinas. West. J. Emerg. Med. 11:247. PMID: 20882144 PMC2941361

[ref35] Kirmayer L. J. Gómez-Carrillo A. (2019). Agency, embodiment and enactment in psychosomatic theory and practice. Med. Humanit. 45, 169–182. doi: 10.1136/medhum-2018-011618, PMID: 31167895 PMC6699606

[ref36] Klein L. B. Chesworth B. R. Howland-Myers J. R. Rizo C. F. Macy R. J. (2021). Housing interventions for intimate partner violence survivors: a systematic review. Trauma Violence Abuse 22, 249–264. doi: 10.1177/15248380198362, PMID: 30913998

[ref37] Lagdon S. Armour C. Stringer M. (2014). Adult experience of mental health outcomes as a result of intimate partner violence victimization: a systematic review. Eur. J. Psychotraumatol. 5:24794. doi: 10.3402/ejpt.v5.24794, PMID: 25279103 PMC4163751

[ref38] Lebre P. Dunphy K. Juma S. (2020). Exploring use of the outcomes framework for dance movement therapy to establish a group profile and objectives for psychomotor therapy interventions. Body Mov. Dance Psychother. 15, 251–266. doi: 10.1080/17432979.2020.1806926

[ref39] Levine P. A.. (2010). In an unspoken voice: How the body releases trauma and restores goodness. Berkeley, CA: North Atlantic Books.

[ref40] Llaurado C. (2008). Observation of the psychomotrician’s intervention: transference attitudes and manifestations. Rev. Interuniv Form Prof. 62, 123–154.

[ref41] Louková T. Hátlová B. Ségard M. (2015). Psychomotor therapy and physical self-concept. Czech Republic: University of JE Purkyně in Ústí nad Labem.

[ref42] Machorrinho J. Veiga G. Santos G. Marmeleira J.. (2021a). Body ownership of women with and without history of intimate-partner violence [poster]. Lisbon: Encontro Ciência.

[ref43] Machorrinho J. Veiga G. Santos G. Marmeleira J. (2021b). Embodiment-related risk factors for posttraumatic stress, anxiety and depression in female victims of intimate-partner violence. J. Trauma Dissociation 2021, 23, 212–228. doi: 10.1080/15299732.2021.1989109, PMID: 34651566

[ref44] Machorrinho J. Veiga G. Santos G. Marmeleira J. (2022). “Embodiment: features, measures and importance in intimate partner violence” in Handbook of anger, aggression and violence. eds. Preedy V. Martin P. (Switzerland: Springer), 1–21.

[ref45] MacLaren H. L.. (2016) *Mutuality in movement: A relational approach to dance/movement therapy with domestic violence survivors*. Unpublished Master’s Thesis, Columbia College Chicago, Chicago, IL.

[ref46] Marcelino D. Gonçalves S. P. (2012). Perturbação pós-stress traumático: Características psicométricas da versão portuguesa da posttraumatic stress disorder checklist–civilian version (PCL-C). Rev. Port Saude Publica 30, 71–75. doi: 10.1016/j.rpsp.2012.03.003

[ref47] Marmeleira J. Machorrinho J. Veiga G. Santos G. (2023). “Psychomotor intervention in intimate partner violence: empirical support for preventive and therapeutic approaches” in Intimate partner violence: Indicators, psychological impact and prevention. ed. Bennett C. (United States: Nova Science Publishers)

[ref48] Neves C. F. Price C. J. Carvalheira A. (2017). The psychometric properties of the scale of body connection (SBC) in a Portuguese sample. Psychol Commun. Health 6:2317. doi: 10.23668/psycharchives.2317

[ref49] Nicholson A. A. Densmore M. Frewen P. A. Théberge J. Neufeld R. W. McKinnon M. C.. (2015). The dissociative subtype of posttraumatic stress disorder: unique resting-state functional connectivity of Basolateral and Centromedial amygdala complexes. Neuropsychopharmacology 40, 2317–2326. doi: 10.1038/npp.2015.79, PMID: 25790021 PMC4538346

[ref50] O’Shea Brown G.. (2021). Dissociation. In healing complex posttraumatic stress disorder: a clinician’s guide. Cham: Springer International Publishing.

[ref51] Ogbe E. Harmon S. Van den Bergh R. Degomme O. (2020). A systematic review of intimate partner violence interventions focused on improving social support and/mental health outcomes of survivors. PLoS One 15:e0235177. doi: 10.1371/journal.pone.0235177, PMID: 32584910 PMC7316294

[ref52] Ogden P. Pain C. Fisher J. (2006). A sensorimotor approach to the treatment of trauma and dissociation. Psychiatr. Clin. N. Am. 29, 263–279. doi: 10.1016/j.psc.2005.10.012, PMID: 16530597

[ref53] Pais-Ribeiro J. Silva I. Ferreira T. Martins A. Meneses R. Baltar M. (2007). Validation study of a Portuguese version of the hospital anxiety and depression scale. Psychol. Health Med. 12, 225–237. doi: 10.1080/13548500500524088, PMID: 17365902

[ref54] Payne P. Levine P. A. Crane-Godreau M. A. (2015). Somatic experiencing: using interoception and proprioception as core elements of trauma therapy. Front. Psychol. 6:93. doi: 10.3389/fpsyg.2015.00093, PMID: 25699005 PMC4316402

[ref55] Polskaya N. A. Melnikova M. A. (2020). Dissociation, trauma and self-harm. Couns. Psychol. Psychother. 28, 25–48. doi: 10.17759/cpp.2020280103

[ref56] Potel C. (2019). Être psychomotricien: Un métier du présent, un métier d’avenir. (Nouvelle édition augmentée). Paris: Érès.

[ref57] Powers M. B. Asmundson G. J. Smits J. A. (2015). Exercise for mood and anxiety disorders: the state-of-the science. Cogn. Behav. Ther. 44, 237–239. doi: 10.1080/16506073.2015.1047286, PMID: 26057087 PMC4545646

[ref58] Price C. (2007). Dissociation reduction in body therapy during sexual abuse recovery. Complement. Ther. Clin. Pract. 13, 116–128. doi: 10.1016/j.ctcp.2006.08.004, PMID: 17400147 PMC1965500

[ref59] Price C. J. Thompson E. A. (2007). Measuring dimensions of body connection: body awareness and bodily dissociation. J. Altern. Complement. Med. 13, 945–953. doi: 10.1089/acm.2007.0537, PMID: 18047441 PMC3029599

[ref60] Price C. J. Thompson E. A. Cheng S. C. (2017). Scale of body connection: a multi-sample construct validation study. PLoS One 12:e0184757. doi: 10.1371/journal.pone.0184757, PMID: 29028803 PMC5640211

[ref61] Price C. J. Wells E. A. Donovan D. M. Rue T. (2012). Mindful awareness in body-oriented therapy as an adjunct to women's substance use disorder treatment: a pilot feasibility study. J. Subst. Abus. Treat. 43, 94–107. doi: 10.1016/j.jsat.2011.09.016, PMID: 22119181 PMC3290748

[ref62] Rekkers M. Scheffers M. van Elburg A. A. Van Busschbach J. T. (2021). The protocol for positive body experience (PBE); introducing a psychomotor therapy intervention based on positive body exposure targeting negative body image in eating disorders. Body Move. Dance Psychother. 16, 252–266. doi: 10.1080/17432979.2020.1863261

[ref63] Rosenbaum S. Sherrington C. Tiedemann A. (2015). Exercise augmentation compared with usual care for post-traumatic stress disorder: a randomized controlled trial. Acta Psychiatr. Scand. 131, 350–359. doi: 10.1111/acps.12371, PMID: 25443996

[ref64] Shepherd L. Wild J. (2014). Emotion regulation, physiological arousal and PTSD symptoms in trauma-exposed individuals. J. Behav. Ther. Exper. Psychiatr. 45, 360–367. doi: 10.1016/j.jbtep.2014.03.002, PMID: 24727342 PMC4053589

[ref65] Snaith R. P. (2003). The hospital anxiety and depression scale. Health Qual. Life Outcomes 1:29. doi: 10.1186/1477-7525-1-29, PMID: 12914662 PMC183845

[ref66] Tavakol M. Dennick R. (2011). Making sense of Cronbach's alpha. Int. J. Med. Educ. 2, 53–55. doi: 10.5116/ijme.4dfb.8dfd, PMID: 28029643 PMC4205511

[ref67] Taylor J. McLean L. Korner A. Stratton E. Glozier N. (2020). Mindfulness and yoga for psychological trauma: systematic review and meta-analysis. J. Trauma Dissoc. 21, 536–573. doi: 10.1080/15299732.2020.1760167, PMID: 32453668

[ref68] Tomczak M. Tomczak E. (2014). The need to report effect size estimates revisited. an overview of some recommended measures of effect size. Trends Sport Sci. 2, 19–25.

[ref69] Tschoeke S. Steinert T. Bichescu-Burian D. (2019). Causal connection between dissociation and ongoing interpersonal violence: a systematic review. Neurosci. Biobehav. Rev. 107, 424–437. doi: 10.1016/j.neubiorev.2019.09.030, PMID: 31562923

[ref70] Van de Kamp M. Hoven M. (2019). “Psychomotor interventions for post-traumatic stress disorder and dissociative disorders” in Psychomotor interventions for mental health–adults. eds. De Lange J. Glas O. Van Busschbach J. Emck C. Scheewe T. (Los Angeles, CA: Boom), 162–179.

[ref71] Van de Kamp M. Scheffers M. Hatzmann J. Emck C. Cuijpers P. Beek P. J. (2019). Body- and movement-oriented interventions for posttraumatic stress disorder: a systematic review and meta-analysis. J. Traumat. Stress 32, 967–976. doi: 10.1002/jts.22465, PMID: 31658401 PMC6973294

[ref72] Van der Kolk B.. (2015). The body keeps the score: Mind, brain and body in the transformation of trauma. United Kingdom: Penguin.

[ref73] Van der Kolk B. (2022). Posttraumatic stress disorder and the nature of trauma. Dialogues Clin. Neurosci. 2, 7–22. doi: 10.31887/DCNS.2000.2.1/bvdkolk, PMID: 22034447 PMC3181584

[ref74] Vaz Serra A. Canavarro M. C. Simões M. Pereira M. Gameiro S. Quartilho M. J.. (2006). Estudos psicométricos do instrumento de avaliação da qualidade de vida da Organização Mundial de Saúde (WHOQOL-Bref) para Português de Portugal. Psiquiat Clín. 27, 41–49. Available at: http://hdl.handle.net/10849/181

[ref75] Weathers F. W. Litz B. Herman D. Juska J. Keane T. (1994). PTSD checklist–civilian version. J. Occup. Health Psychol. 1:2622. doi: 10.1037/t02622-000

[ref76] Weaver T. L. Resnick H. (2004). Toward developing complex multivariate models for examining the intimate partner violence-physical health relationship. J. Interper. Viol. 19, 1342–1349. doi: 10.1177/0886260504269692, PMID: 15534336

[ref77] World Health Organization. (2021). *Violence against women*. Geneva: World Health Organization. Available at: https://www.who.int/news-room/fact-sheets/detail/violence-against-women. (Accessed October 10, 2022).

[ref78] Yakubovich A. R. Bartsch A. Metheny N. Gesink D. O’Campo P. (2022). Housing interventions for women experiencing intimate partner violence: a systematic review. Lancet 7, e23–e35. doi: 10.1016/S2468-2667(21)00234-6, PMID: 34838218

[ref79] You S. Talbot N. L. He H. Conner K. R. (2012). Emotions and suicidal ideation among depressed women with childhood sexual abuse histories. Suicide Life Threat. Behav. 42, 244–254. doi: 10.1111/j.1943-278X.2012.00086.x, PMID: 22409700 PMC3406484

[ref80] Zamir O. Szepsenwol O. Englund M. M. Simpson J. A. (2018). The role of dissociation in revictimization across the lifespan: a 32-year prospective study. Child Abuse Negl. 79, 144–153. doi: 10.1016/j.chiabu.2018.02.001, PMID: 29454258

[ref81] Zigmond A. S. Snaith R. P. (1983). The hospital and anxiety and depression scale. Acta Psychiatr. Scand. 67, 361–370. doi: 10.1111/j.1600-0447.1983.tb09716.x 6880820

